# Case report: A multiple sclerosis patient with imaging features of glymphatic failure benefitted from CSF flow shunting

**DOI:** 10.3389/fnins.2022.863117

**Published:** 2022-11-01

**Authors:** Antonio Scollato, Francesco Lolli, Giancarlo Lastrucci, Anna Repice, Giuseppe De Santis, Claudio Nicoletti, Berardino Porfirio, Pasquale Gallina

**Affiliations:** ^1^Neurosurgery Unit, “Cardinale Panico” Hospital, Tricase, Italy; ^2^Department of Clinical and Experimental Biomedical Sciences “Mario Serio”, University of Florence, Florence, Italy; ^3^Neurophysiology Unit, Careggi University Hospital, Florence, Italy; ^4^Department of Neurosciences, Psychology, Drug Research and Child Health, University of Florence, Florence, Italy; ^5^Neurology Unit, Careggi University Hospital, Florence, Italy; ^6^Neurology Unit, “Cardinale Panico” Hospital, Tricase, Italy; ^7^Department of Experimental and Clinical Medicine, Section of Anatomy, University of Florence, Florence, Italy; ^8^Neurosurgery Unit, Careggi University Hospital, Florence, Italy

**Keywords:** glymphatic system, multiple sclerosis, perivascular space, CSF dynamic disturbances, CSF shunting

## Abstract

The derangement of CSF circulation impacts the functions of the glymphatic-lymphatic system (G-Ls), which regulates solute trafficking and immune surveillance in the CNS. The G-Ls failure leads to the dysregulation of clearance of waste molecules in the brain and to an altered CNS immune response. The imaging features of dilated perivascular spaces imply the impairment of the G-Ls. We report on the case of a patient with primary progressive multiple sclerosis and dilatation of perivascular spaces, who transiently improved after CSF shunt diversions. The underlying mechanisms remain to be determined and at this stage, it is not possible to link CSF diversion to an effect on MS pathology. However, this observation provides the rationale to incentivize research in the largely unknown area of CSF dynamic disturbances on G-Ls failure and ultimately in neurodegeneration.

## Introduction

The glymphatic-lymphatic system (G-Ls) regulates solute trafficking and immune surveillance in the CNS, sub-serving the flow of CSF from the subarachnoid spaces into the perivascular spaces and subsequently into the interstitium, with the aquaporin-4 water channels running it (Iliff et al., 2012; Louveau et al., 2018; Nedergaard and Goldman, 2020; Mestre et al., 2022). The cerebrospinal-interstitial fluid then passes to the venous perivascular and perineural spaces, lastly draining toxic molecules and immune cells from the brain into meninges and deep lymph nodes.

Derangements in intra-extracranial hydrodynamics at various levels lead to the failure of the CSF dynamic, resulting in G-Ls failure (Nedergaard and Goldman, 2020), which possibly configures “G-Ls pathology” (Mestre et al., 2022). The characteristic imaging pattern of G-Ls dysfunction is perivascular space dilatation caused by internal congestion and stagnation with an accumulation of cerebrospinal-interstitial fluid (see Wardlaw et al., 2020 and references within).

The glymphatic-lymphatic system is impaired in multiple sclerosis (MS) (Carotenuto et al., 2021). Dilated perivascular spaces occur more frequently in patients with MS than in controls (Wuerfel et al., 2008; Etemadifar et al., 2011; Kilsdonk et al., 2015; Granberg et al., 2020; Wardlaw et al., 2020). In an experimental model of autoimmune encephalitis, perivascular aquaporin-4 localization is lost (Wolburg-Buchholz et al., 2009). Inflammation disrupts the association of astrocytes with blood vessels and surrounding neurons in MS (Eilam et al., 2018). Inflammatory follicle-like aggregates in progressive MS are located in these perivascular spaces (Lassmann, 2018). Taken together these findings suggest that improving G-Ls flow in an MS patient with perivascular space dilatation, forcing CSF circulation through the diversion as described in other models (Abolfazli et al., 2016), might contribute to the clearance of inflammatory mediators in MS (Khaibullin et al., 2017), and thus potentially improving the clinical picture.

## Case presentation

**Figure 1:**
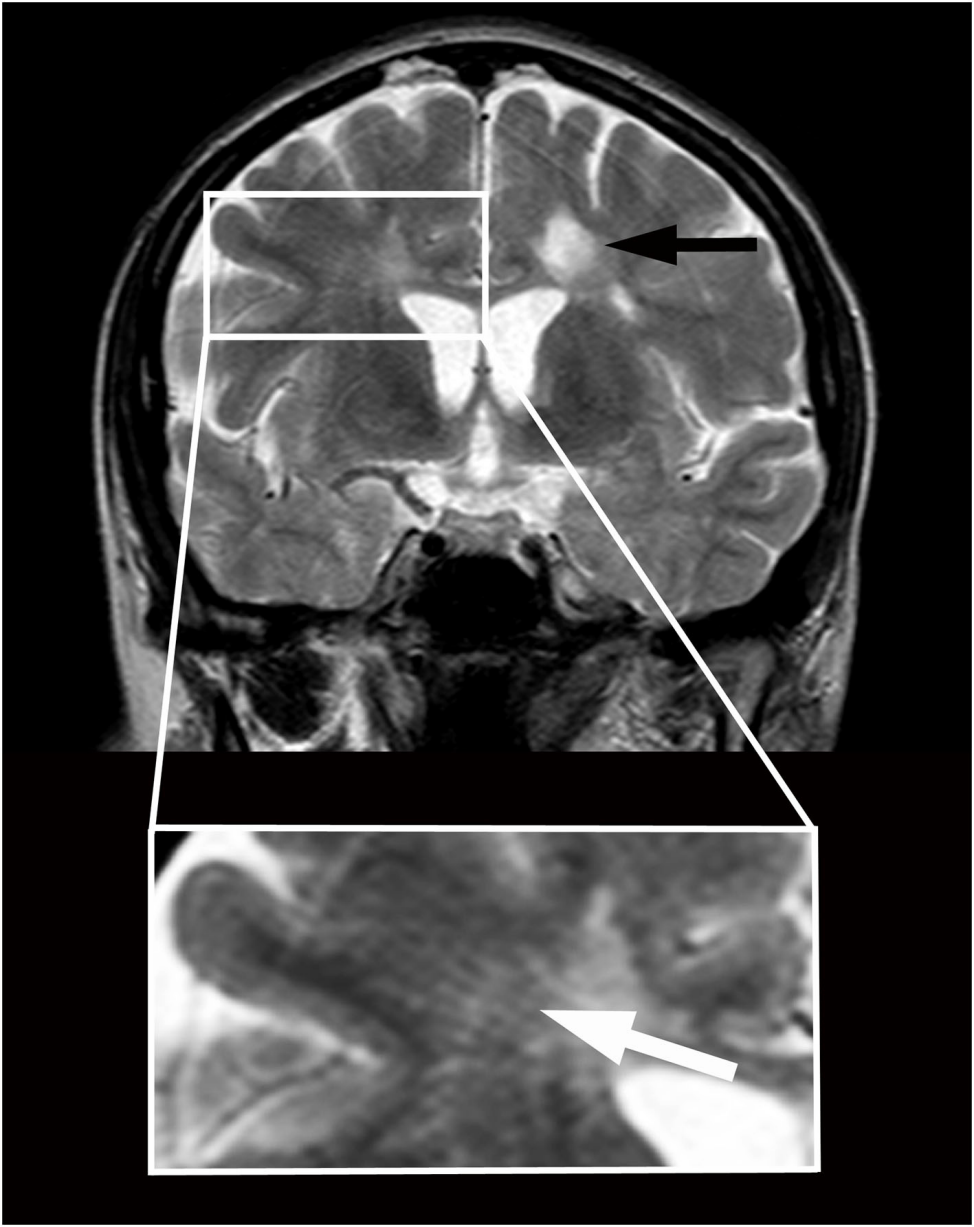
Magnetic resonance imaging in a patient with primary progressive multiple sclerosis and features of brain interstitial space congestion. Imaging was performed before positioning a lumboperitoneal shunt. Multiple sclerosis lesions appear as round hyperintensities on coronary T2-weighted imaging obtained at the level of the third ventricle (black arrow). Enlarged perivascular spaces appear as linear hyperintensities crossing radially the Corona Radiata from the cortex to the ventricles (white arrow in higher magnification below). The size of the ventricles is normal.

In 2009, a 33-year-old woman progressively experienced the onset of headaches, gait disturbances, and urinary dysfunction. Fluid-attenuated inversion recovery images of the brain magnetic resonance imaging (MRI), performed in 2010, revealed diffuse hyperintense T2 nodular lesions affecting the paraventricular and subcortical white matter; at least two of these lesions showed gadolinium enhancement in T1-weighted images. A coeval MRI of the spinal cord showed nodular lesions involving the dorsal columns, at the C2 and C3 levels. The blood tests were within physiological parameters, while the neurophysiological examination revealed changes in visual evoked potential and somatosensory evoked potential from the left median nerve. Oligoclonal IgG bands at isoelectric focusing occurred in the CSF. The clinical and paraclinical findings led to the diagnosis of MS. Methylprednisolone provided the patient with some improvement. Immunomodulatory treatments with interferon-beta and glatiramer acetate were started and later discontinued due to intolerance. After 16 months of continuous neurological impairment, the patient was diagnosed with primary progressive MS. An MRI performed during the same period revealed a stable picture. She was voluntarily tested in another hospital for cerebrospinal venous insufficiency in 2012. A severe cerebrospinal venous insufficiency was diagnosed, and she underwent percutaneous transluminal angioplasty at the level of the internal jugular veins. She had about 2 years of clinical benefit after the procedure, which paralleled normal anatomic and functional conditions of jugular veins according to the echo color Doppler examination and stability of MS lesions at MRI. Subsequently, her neurological condition deteriorated gradually. Sonographic findings suggested a recurrence of chronic cerebrospinal venous insufficiency (2014). At the age of 38, the patient came under our observation (Supplementary Video 1) with a disease duration of 5 years. Her Kurtzke Expanded Disability Status Scale (EDSS) was 6.5. T2-weighted brain MRI (Figure 1) performed in 2014 showed enlarged perivascular spaces as linear-, ovoid-, or round-shaped (depending on the slice direction) hyperintensities (Adams et al., 2013), associated with typical MS lesions (Wuerfel et al., 2008). Considering the severe progression of the disease, the patient gave consent to undergo a program of CSF diversions as compassionate treatment. She received two 1-day external lumbar drainages (12–15 ml/h over 24 h) (Gallina et al., 2018). At the end of the drainages, the patient experienced marked clinical improvement (Supplementary Video 2) and EDSS 3.0 a week after both procedures. This status lasted about 2 months after the first drainage and about 1 month after the second one. Her headaches, in particular, vanished, and she regained her gait and urinary functions. In August 2014, signs/symptoms progressively reappeared in association with mild cognitive impairment (Mini-Mental State Examination score of 25), and the EDSS score reached 8.0. MRI detected a dimensional increase of the subcortical right frontal MS lesion. In September 2014, the patient underwent lumboperitoneal shunt (Spetzler system, Integra LifeSciences Corp) implantation. Once mobilized (about 24 h after the intervention), she showed mild spastic-ataxic paraparesis with full autonomy in gait, sphincter recovery, and headache resolution. An MRI performed in October 2014 revealed the absence of contrast enhancement of the brain lesions. Further improvement was progressively observed (Supplementary Video 3), but a slow worsening occurred 5 months after surgery. One year after the lumboperitoneal drainage, an MRI study revealed the absence of active MS lesions and the persistence of perivascular space enlargement. Thirteen months after surgery, she received an EDSS score of 7.5. An ophthalmological examination revealed severe right optic impairment. The patient refused further external lumbar drainage to verify the hypothesis that clinical worsening was due to shunt failure. In October 2018, her MRI was stable, with no signs of activity, and her neurological conditions remained unchanged.

## Discussion

Aware of the anecdotal nature, potential placebo effect, and possibly unrelated and unknown factors, this case suggests the effect of CSF diversions on the MS patient's clinical improvements. The mechanisms and factors underpinning the CSF shunt-mediated improvements of the MS symptoms remain to be determined, at this stage. CSF diversion, decongesting perivascular spaces, may have ameliorated glymphatic functioning and interstitial removal of proinflammatory molecules from CSF and in so doing, downregulating local inflammation. This is in line with the Louveau et al. hypothesis that the modulation of CSF drainage through the meningeal lymphatic vasculature might reduce the quantity of CNS antigens entering the related lymph nodes, resulting in the downregulation of the autoimmune response (Louveau et al., 2016). Measurement of inflammatory markers before and after CSF diversions would have clarified if the improved clearance of inflammatory mediators underlain the clinical changes in our patients. The observation that perivascular spaces remained enlarged after CSF diversion is not surprising; indeed, post-inflammatory fibrosis might make perivascular enlargement a permanent feature (Inglese et al., 2005).

However, the hypothesis of a “hydraulic” mechanism related to changes in intracranial hydrodynamics after CSF subtraction, as responsible/co-responsible for neurological improvement cannot be excluded. Notably, the dominant signs/symptoms in our patient, i.e., gait and urinary disturbances, cognitive deterioration, and headache are also characteristic of normal pressure hydrocephalus (Adams et al., 1965). As in this latter condition, related to a failure of CSF dynamics condition, clinical disturbances quickly improved after lumbar drainages, which involve a momentary reduction of CSF volume (Gallina et al., 2018), and slowly vanished due to CSF volume restoring, once drains were removed. Moreover, it is unclear why the benefit observed in our patient following definitive CSF diversion was limited to months. Following insufficiency/stop of CSF flux drainage due to shunt failure is one possibility.

This report does not suggest a new therapy for MS. It represents an exploratory approach to neurodegenerative diseases (Scollato et al., 2016), in which CSF flow disturbances play a possible role in the failure of G-Ls (Nedergaard and Goldman, 2020; Gallina et al., 2021), leading to inadequate brain clearance of toxic molecules and immune cells (Iliff et al., 2012).

## Data availability statement

The original contributions presented in the study are included in the article/Supplementary material, further inquiries can be directed to the corresponding author/s.

## Ethics statement

Ethical review and approval were not required for the study on human participants in accordance with the local legislation and institutional requirements. The patient gave consent to undergo the therapeutic program as compassionate treatment. Written informed consent was obtained from the individual(s) for the publication of any potentially identifiable images or data included in this article.

## Author contributions

AS, BP, and PG have made substantial contribution to the conception of the study. PG, FL, BP, AR, GL, CN, and GD have drafted the work or substantially revised it. All authors contributed to the article and approved the submitted version.

## Conflict of interest

The authors declare that the research was conducted in the absence of any commercial or financial relationships that could be construed as a potential conflict of interest.

## Publisher's note

All claims expressed in this article are solely those of the authors and do not necessarily represent those of their affiliated organizations, or those of the publisher, the editors and the reviewers. Any product that may be evaluated in this article, or claim that may be made by its manufacturer, is not guaranteed or endorsed by the publisher.

Videos of a patient with primary progressive multiple sclerosis imaging features of dilated perivascular spaces who underwent cerebrospinal fluid diversions.

## Supplementary material

Supplementary Video 1 Video obtained before external lumbar drainage positioning demonstrating severe difficulty in walking.

Supplementary Video 2 Video obtained within 1 h after external lumbar drainage removal showing a striking improvement of gait.

Supplementary Video 3 Video obtained 3 months after lumboperitoneal shunting, showing normal gait and equilibrium.
